# Initiation of ventricular arrhythmia in the acquired long QT syndrome

**DOI:** 10.1093/cvr/cvac103

**Published:** 2022-06-21

**Authors:** Cherry Alexander, Martin J Bishop, Rebecca J Gilchrist, Francis L Burton, Godfrey L Smith, Rachel C Myles

**Affiliations:** Institute of Cardiovascular and Medical Sciences, University of Glasgow, Glasgow, G12 8QQ, UK; School of Biomedical Engineering and Imaging Sciences, King’s College London, St Thomas' Hospital, Westminster Bridge Road, London SE1 7EH, UK; Institute of Cardiovascular and Medical Sciences, University of Glasgow, Glasgow, G12 8QQ, UK; Institute of Cardiovascular and Medical Sciences, University of Glasgow, Glasgow, G12 8QQ, UK; Institute of Cardiovascular and Medical Sciences, University of Glasgow, Glasgow, G12 8QQ, UK; Institute of Cardiovascular and Medical Sciences, University of Glasgow, Glasgow, G12 8QQ, UK

**Keywords:** Electrophysiology, Ventricular arrhythmia, Long QT syndrome, Optical mapping

## Abstract

**Aims:**

Long QT syndrome (LQTS) carries a risk of life-threatening polymorphic ventricular tachycardia (Torsades de Pointes, TdP) and is a major cause of premature sudden cardiac death. TdP is induced by R-on-T premature ventricular complexes (PVCs), thought to be generated by cellular early-afterdepolarisations (EADs). However, EADs in tissue require cellular synchronisation, and their role in TdP induction remains unclear. We aimed to determine the mechanism of TdP induction in rabbit hearts with acquired LQTS (aLQTS).

**Methods and results:**

Optical mapping of action potentials (APs) and intracellular Ca^2+^ was performed in Langendorff-perfused rabbit hearts (*n* = 17). TdP induced by R-on-T PVCs was observed during aLQTS (50% K^+^/Mg^++^ & E4031) conditions in all hearts (*P* < 0.0001 vs. control). Islands of AP prolongation bounded by steep voltage gradients (VGs) were consistently observed before arrhythmia and peak VGs were more closely related to the PVC upstroke than EADs, both temporally (7 ± 5 ms vs. 44 ± 27 ms, *P* < 0.0001) and spatially (1.0 ± 0.7 vs. 3.6 ± 0.9 mm, *P* < 0.0001). PVCs were initiated at estimated voltages of ∼ −40 mV and had upstroke dF/dt_max_ and V_m_-Ca^2+^ dynamics compatible with *I*_CaL_ activation. Computational simulations demonstrated that PVCs could arise directly from VGs, through electrotonic triggering of *I*_CaL_. In experiments and the model, sub-maximal L-type Ca^2+^ channel (LTCC) block (200 nM nifedipine and 90% gCaL, respectively) abolished both PVCs and TdP in the continued presence of aLQTS.

**Conclusion:**

These data demonstrate that *I*_CaL_ activation at sites displaying steep VGs generates the PVCs which induce TdP, providing a mechanism and rationale for LTCC blockers as a novel therapeutic approach in LQTS.

## Graphical Abstract

**Graphical abstract:**
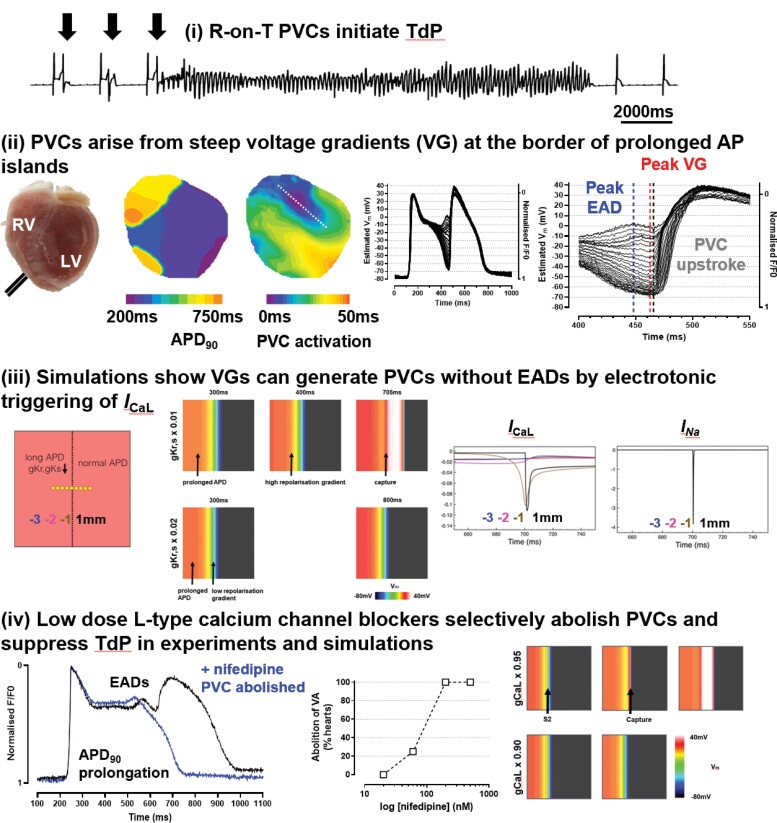
(i) Pseudo-ECG recorded from a Langendorff-perfused rabbit heart under aLQTS conditions during pacing at 2000ms showing induction of TdP by spontaneous PVCs. (ii) Contour maps showing APD90 for a paced beat and AT of the subsequent PVC with contiguous single-pixel AP traces taken from dashed line plotted with estVm and expanded to show the temporal relationship between the peak of the EAD (blue dashed line), the peak VG (red dashed line) and the earliest upstroke of the PVC (dark grey dashed line). (iii) Simulations of the border of a prolonged AP island, showing the initiation of a PVC at the border of the long APD region with propagation into the normal APD region with ICaL and INa traces sampled at 1mm spacing from the border. (iv) Superimposed AP traces showing abolition of PVCs with nifedipine in the presence of EADs and APD prolongation, with dose-response for anti-arrhythmic effect of nifedipine and recapitulation with reduction of gCaL in simulations.

**Time of primary review: 43 days See the editorial comment for this article ‘A unifying mechanism for the initiation of torsade de pointes: blurring the distinction between trigger and substrate’, by M. McKay and F. G. Akar, https://doi.org/10.1093/cvr/cvad033.**

## 1. Introduction

The long QT syndrome (LQTS) is associated with a risk of life-threatening ventricular arrhythmias (VAs) and sudden cardiac death (SCD).^1^ QT prolongation is linked to Torsades de Pointes (TdP), a specific form of polymorphic ventricular tachycardia which causes rapid haemodynamic compromise and can degenerate to ventricular fibrillation.^2^ The key pathogenic feature of LQTS is delayed repolarisation, resulting in prolongation of the cardiac action potential (AP), which manifests as QT interval (QTc) prolongation on the surface electrocardiogram (ECG). LQTS may be inherited (iLQTS) or acquired (aLQTS), and the first presentation may be with SCD or resuscitated cardiac arrest.^3^ The majority of iLQTS (∼75%) is accounted for by loss-of-function mutations in *I*_Ks_ (LQT1),^4^*I*_Kr_ (LQT2)^5^ or gain-of-function mutations in *I*_Na_ (LQT3).^6^ aLQTS is generally caused by pharmacological block of the *I*_Kr_ channel.^2^ In both, risk modifiers including bradycardia, electrolyte imbalance and sympathetic activation can lead to dynamic changes in QTc and TdP risk.^3,7^ Regardless of the cause of QTc prolongation, TdP is initiated through a final common pathway.^8^ A focally-triggered premature ventricular complex (PVC) interacts with a vulnerable substrate to produce re-entrant activation and sustained arrhythmia.^2^ In some cases, triggered activity (TA) is repetitive, producing bursts or non-sustained TdP, which are often a precursor to sustained re-entrant TdP.^9–11^

While TdP risk increases with increasing QTc, the relationship is insufficiently specific to allow accurate risk prediction.^12^ Beta-blockers are the only guideline-recommended pharmacological treatment,^13^ although some progress is being made with genotype-specific therapy.^14^ The implantable cardioverter-defibrillator (ICD) is highly effective in preventing arrhythmic SCD.^15^ However, the associated complications and morbidity mean that ICDs are not suitable for widespread use in patients with heterogeneous risk profiles.^16^ Novel treatment approaches to reduce SCD risk in LQTS are urgently needed.

Work in isolated cardiomyocytes has demonstrated that AP prolongation predisposes to early-afterdepolarisations (EADs).^17–20^ These abnormal depolarisations occur during the plateau phase and, if of sufficient magnitude, can trigger regenerative currents which support premature APs. Although this mechanism has never been demonstrated directly in whole-heart experiments, the assumption of a causal association between cellular EADs and the PVCs that initiate TdP has meant that therapeutic targets in LQTS have been AP/QTc prolongation and/or EADs.

Many experimental studies have examined the occurrence of EADs and TdP induction in the intact heart.^21–25^ Computational modelling studies have outlined the spatiotemporal synchronization required for cellular EADs to overcome the electrotonic source–sink mismatch and initiate a PVC in coupled tissue.^26^ However, the specific mechanism(s) by which cellular EADs could be synchronized to form tissue-level EADs remain unknown.^27,28^ Recent *in silico* studies have also highlighted the importance of voltage gradients (VGs) in generating PVCs under LQTS conditions.^10,25,29^

We sought to examine the initiation mechanism of focal R-on-T PVCs in a whole-heart model of LQTS using high-resolution optical mapping to determine the relationship between synchronized EADs, VGs and triggered APs in tissue. We employed complementary computational models to test PVC induction mechanisms and identify anti-arrhythmic strategies, which we then assessed in experiments and simulations.

## 2. Methods

### 2.1 Experimental studies

#### 2.1.1 Langendorff-perfused rabbit hearts

All procedures involving animals were undertaken per the UK Animals (Scientific Procedures) Act 1986 under Project Licence PP5254544. Male New Zealand White rabbits (*n* = 17, Envigo UK) were anaesthetized with an intravenous injection of pentobarbital sodium (50 mg/kg) containing 1000 IU of heparin, hearts were rapidly excised and Langendorff-perfused at 37°C. Atrioventricular nodal conduction was ablated by injection of 10% formalin (0.01–0.02 mLs).

#### 2.1.2 Dual optical mapping of voltage and calcium

Hearts were loaded with the fluorescent intracellular Ca^2+^ ([Ca]_i_) indicator Rhod-2AM and the voltage-sensitive dye RH237. Blebbistatin (10µmol/L) was used to eliminate motion artefacts during wide-field optical imaging of epicardial transmembrane potential (V_m_) and [Ca]_i_ using a dual Micam-Ultima complementary metal–oxide semiconductor camera system on a THT macroscope (SciMedia, Costa Mesa, CA).

#### 2.1.3 Experimental protocols

See Supplementary material online, *Figure S1*. Optical recordings were taken during ventricular pacing at cycle lengths (CLs) of 350 ms/2000 ms and in intrinsic rhythm. Pharmacological LQT was induced by switching to Tyrodes’ solution containing 50% K^+^/Mg^2+^ and 0.5µM E4031 (*n* = 13). In 11 experiments, the effects of nifedipine were studied (200 nM, *n* = 6; 500 nM, *n* = 5). In 4 of 11, a dose-response was performed (20, 60, 200 nM). In 5 of 11, E4031 concentration was increased (2 µM) in the presence of nifedipine.

#### 2.1.4 Data analysis

The occurrence of VA, including PVCs, bursts (2–5 PVCs) and TdP (>5 consecutive ventricular beats of varying morphology^30^) was quantified from pseudo-ECGs. Optical data analysis was performed using custom software (*Optiq*, Dr Francis Burton) as previously described.^31^ Epicardial dispersion of AP duration at 90% repolarisation (APD_90_) was defined as the 5–95% range. The rate of rise during the PVC upstroke was defined as dF/dt_max_ of the V_m_ signal expressed as a percentage of the preceding AP. For PVCs, we calculated the earliest activation time (AT) relative to the QRS. Where the earliest epicardial AT was ≥20 ms pre-QRS, initiation close to the epicardial surface was considered likely, and the initiation of these PVCs was analysed in detail. Consecutive single-pixel traces were exported, and fluorescence (F) intensity was normalized to baseline (F0). Based on microelectrode recordings in isolated rabbit epicardial cells,^32^ we estimated that the AP upstroke would span −80 to +30 mV and mapped F/F0 to this scale to give an estimated V_m_ (estV_m_). VGs across sampled traces were quantified as estimated mV/pixel. The peak EAD was defined as pixel and frame with the highest normalized F value during an EAD. The PVC upstroke was defined as the pixel and frame with the earliest positive deflection in normalized F during the initiation of the PVC.

Continuous variables are presented as mean ± standard deviation. Comparisons between two groups of continuous data were made using a Student’s *t*-test, paired where appropriate, and for categorical data using Fisher’s exact test. Comparisons between three or more groups were made using one-way analysis of variance (ANOVA), repeated measures where appropriate, with Bonferroni’s post-testing for multiple comparisons. *P* < 0.05 was considered statistically significant.

### 2.2 Computational modelling studies

*In silico* investigations were performed to dissect the specific mechanisms of the experimentally witnessed phenomena.

#### 2.2.1 Model set-up

A monodomain representation of cardiac electrophysiology was used to simulate pacing protocols over a 2D sheet of cardiac tissue of dimensions 20 mm × 20 mm with fibre orientation in the *x*-direction (Supplementary material online, *Figure S2A*). The sheet was discretized into a triangular finite element mesh of resolution 200 µm. Cellular ionic dynamics were represented by the general mammalian LuoRudyII cell model,^33^ with tissue conductivity initially assigned to experimentally determined values.^34^ Simulations were performed with the Cardiac Arrhythmia Research Package.^35^

#### 2.2.2 Electrophysiological properties

To replicate the experimentally recorded long APD islands, the left-half of the tissue was assigned a prolonged APD by modulating the maximum conductances of *I*_Kr_ and *I*_Ks_ (Supplementary material online, *Figure S2A*). To control the repolarisation gradient formed between the long APD region (left) and the normal APD region (right), tissue conductivity was globally modulated (Supplementary material online, *Figure S2B*). Different combinations of prolonged APD and modulated tissue conductivity were used throughout.

#### 2.2.3 Pacing protocol

The model was paced 100 times to reach the steady state. A single S1 beat was then simulated by pacing the entire lefthand edge of the tissue to initiate a planar wave propagating left-to-right. In a subset of simulations, an additional S2 was prescribed at −4 mm from the vertical tissue centre line (within the region of long APD), stimulating a 200 μm-thin vertical strip of tissue, as shown in Supplementary material online, *Figure S2C*. The S2 stimulus was delivered at 500 ms following the S1 at threshold strength for 2 ms duration. This S2 represented a pseudo-EAD delivered at a controlled timing and location, facilitating a direct comparison between different subsequent simulations.

#### 2.2.4 Data analysis

V_m_, [Ca]_i_, fast sodium current (*I*_Na_) and the L-type calcium current (*I*_CaL_) were analysed from a series of points located across the centre line separating the long- and normal APD regions (Supplementary material online, *Figure S2B*).

## 3. Results

### 3.1 Focal triggering of VAs in pharmacological LQTS

**Figure 1:**
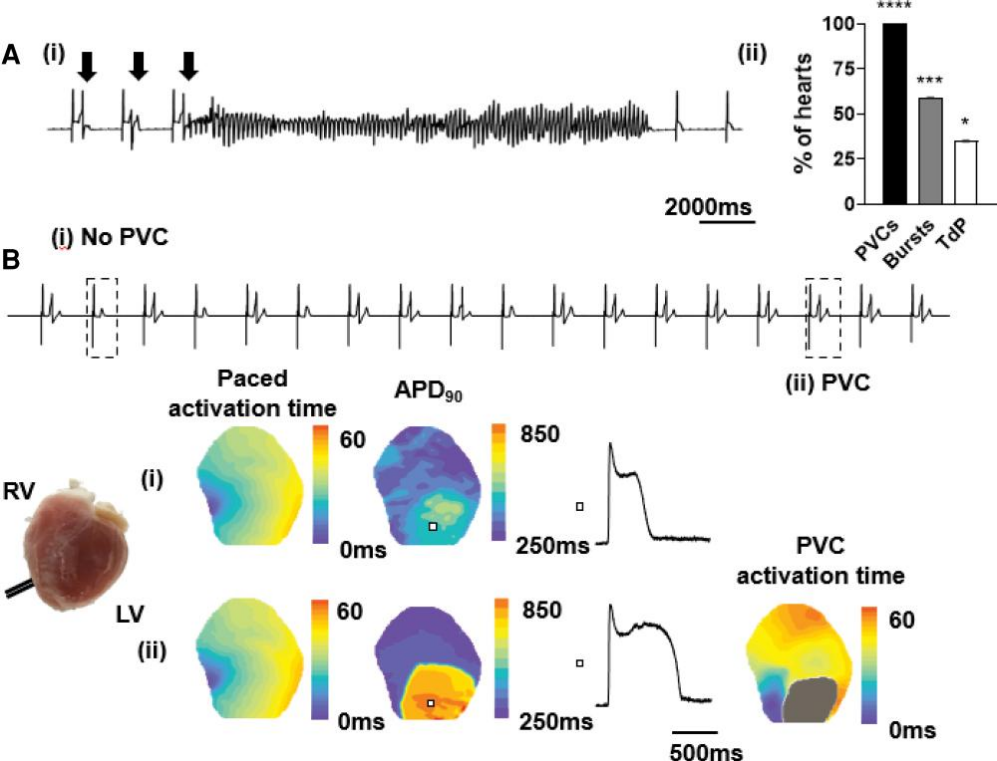
Ventricular arrhythmia in rabbit hearts with aLQTS. (*A*) (i) Pseudo-ECG recorded from a Langendorff-perfused rabbit heart under aLQTS conditions during pacing at 2000 ms showing induction of TdP by spontaneous PVCs (arrows), (ii) occurrence of PVCs, bursts (2–5 beats) and TdP (*n* = 17), Fisher’s exact test vs. control, ****P < 0.0001, ***P < 0.001, *P < 0.05. (*B*) pECG showing paced beats (i) without and (ii) with PVCs. Contour maps showing AT and APD_90_ for beats (i) and (ii), with AP traces from the area indicated. The AT map for the PVC is also shown in (ii) and demonstrates the PVC arising from the border of the long APD island.

An acute pharmacological model of aLQTS was induced in isolated perfused rabbit hearts using a combination of *I*_Kr_ blockade and 50% K^+^/Mg^2+^ (*Figure 1*), resulting in rate-dependent APD prolongation (Supplementary material online, *Table*SI). At CLs ≥2000 ms, focal R-on-T PVCs occurred in all hearts under aLQTS conditions (*P* < 0.0001 vs. control, *n* = 17, *Figure 1Aii*). Bursts occurred in 10 of 17 hearts (59%, *P* < 0.001 vs. control) and TdP in 6 of 17 hearts (35%, *P* < 0.05 vs. control). For optically mapped R-on-T PVCs (*n* = 238 in 17 hearts), the earliest epicardial V_m_ AT relative to the QRS ranged from 48 ms pre-QRS to 49 ms post-QRS. 31 PVCs mapped (13%) in 13 of 17 hearts had an earliest epicardial V_m_ AT ≥20 ms pre-QRS and were defined as epicardial-origin PVCs.

### 3.2 Electrotonic triggering of focal PVCs in areas of steep VGs

**Figure 2:**
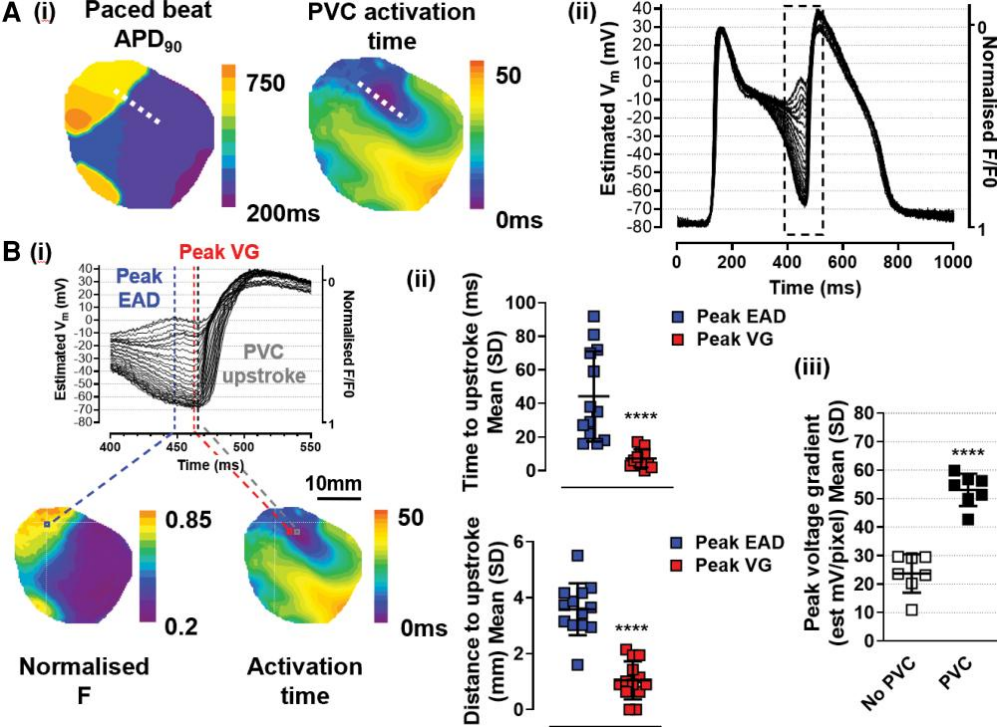
Role of EADs and VGs in PVC initiation. (*A*) (i) Contour maps showing APD_90_ for a paced beat and AT of the subsequent PVC and (ii) contiguous single-pixel AP traces taken from dashed line in (i) plotted with estV_m_. (*B*) (i) detail from A(ii) expanded to show the temporal relationship between the peak of the EAD (blue dashed line), the peak VG (red dashed line) and the earliest upstroke of the PVC (dark grey dashed line), the spatial separation is shown in the contour maps of normalized fluorescence (F) and AT below (ii) mean data (*n* = 13 PVCs from 11 hearts) showing time and distance to PVC upstroke from the peak EAD (blue symbols) and the peak VG (red symbols), (iii) mean data (*n* = 7 PVCs in 5 hearts) showing peak VG in beats without a PVC (open symbols) and subsequent beat with a PVC (closed symbols). Paired Student’s *t*-tests, **** *P* < 0.0001.

A typical example of epicardial R-on-T PVC induction under aLQTS conditions is shown in *Figure 1B*. In all 31 epicardial-origin PVCs, we observed ‘islands’ of APD prolongation bordered by steep repolarisation VGs. The size and location of these islands is described in Supplementary material online, *Figure S3*. PVCs consistently arose from the border between the long and normal APD regions. Tissue-level EADs were manifest as positive V_m_ deflections during the AP plateau. In most cases, PVC initiation was associated with a local tissue-level phase 2 EAD [13/17 PVCs (76%) under LQTS conditions in 11/11 hearts]. In the remaining four cases, the initiation site was near the edge of the mapped region, and local EADs may have been present outside the mapping window. To determine the role of EADs in PVC initiation, the peak of the EAD and the earliest upstroke of the PVC were compared (*Figure 2*). The peak EAD preceded the PVC upstroke by 44 ± 27 ms and occurred at distance from the PVC upstroke of 3.6 ± 0.9 mm (*Figure 1Bii*). The estV_m_ range was significantly different (peak EAD 4.7 ± 7.2 mV vs. PVC upstroke −39.1 ± 5.1 mV, *P* < 0.0001). EADs occurring at the border of the prolonged APD region produced a dynamic increase in local VGs (*Figure 2Aii*). As shown in *Figure 2B*, the peak VG was more closely associated with the upstroke of the PVC than was the EAD, both temporally (*Figure 1Bii*, 7 ± 5 ms vs. 44 ± 27 ms, *P* < 0.0001) and spatially (*Figure 1Bii*, 1.0 ± 0.7 vs. 3.6 ± 0.9 mm, *P* < 0.0001). Additionally, paced beats associated with PVCs displayed significantly larger VGs than preceding beats, which did not support a PVC (*Figure 2Biii*, 53.1 ± 5.7 vs. 23.7 ± 6.8 estimated mV/pixel, *P* < 0.0001). A qualitative comparison of plateau V_m_ profiles between consecutive beats with and without a PVC suggested that both a local EAD and earlier repolarisation in adjacent sites contributed to the VG (Supplementary material online, *Figure S4*).

### 3.3 VGs alone can trigger ectopic beats

These features suggest that steep VGs were responsible for the induction of PVCs. The EADs did not directly trigger PVC initiation but instead were one of the factors that increased VGs when they occurred close to the border of the prolonged APD region. We were not able to separate EADs and steep VGs using experimental manipulations. Therefore, we used a computational model to test our hypothesis that steep VGs, not EADs, were necessary to initiate R-on-T PVCs. In 2D simulations of steep VGs, PVC initiation occurred in the absence of EADs (Supplementary material online, *Figure S5*). Spontaneous PVC formation was seen in the presence of a VG >33.7 mV/mm, maintained by imposing a reduction of ×0.01 to repolarising potassium currents in the long APD region (Supplementary material online, *Figure S5Ai*). Reducing *I*_Kr_ and *I*_Ks_ by ×0.02 in the long APD region reduced the VG (27.2 mV/mm), and no spontaneous PVC was seen (Supplementary material online, *Figure S5Aii*). These data support the conclusion that electrotonic triggering by steep VGs in tissue is the mechanism for the PVC initiation we observed experimentally. In the simulations, the introduction of pseudo-EADs near the border of the prolonged APD region (applied as a weak S2 at 500 ms, as shown in Supplementary material online, *Figure S2B*) made PVC initiation at the boundary more likely, in keeping with the experimental observation that EADs increased local VGs and were associated with electrotonic triggering.

### 3.4 Evidence for LTCC activation during electrotonic triggering

**Figure 3:**
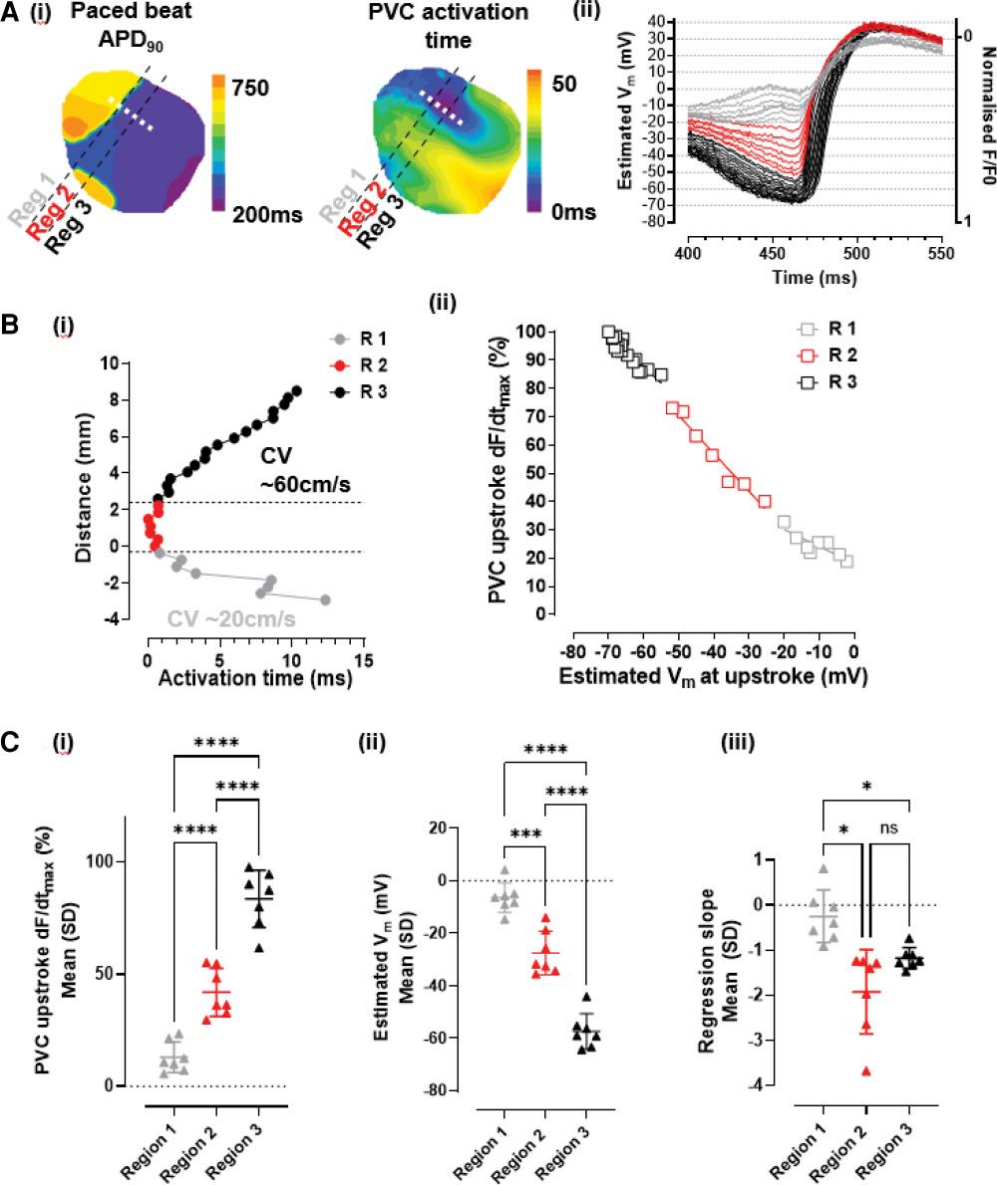
PVC upstroke characteristics. (*A*) (i) Contour maps showing APD_90_ for a paced beat and AT from the same example given in *Figure 2*. Three regions are indicated, region 1 – long APD island, region 2 – early activated region and region 3 – late activated region (ii) contiguous single-pixel AP upstroke traces taken from white dashed line indicated in (i) and plotted with estV_m_, the trace colours denote the region, region 1 (grey), region 2 (red), and region 3 (black). (*B*) (i) Distance–time plot showing synchronous activation in region 2 (R2) and CV estimates for regions 1 (R1) and 3 (R3) (ii) correlation between upstroke dF/dt_max_ and estV_m_ at upstroke for regions 1–3. (*C*) Average regional values for (i) PVC upstroke dF/dt_max_ (ii) estV_m_ and (iii) regression slope for correlation between upstroke dF/dt_max_ and estV_m_ for epicardial PVC initiation in 7 hearts (RM one-way ANOVA, *P < 0.05, ***P < 0.001, ****P < 0.0001).

Next, we examined AP upstrokes and V_m_-Ca^2+^ dynamics during epicardial R-on-T PVCs, a representative example of which is shown in *Figure 3A and B*. Distance–time plots delineated three regions with distinct activation profiles: Region 1, the prolonged AP region, Region 2, the early activated region during the PVC, and Region 3, PVC propagation. As shown in *Figure 3C*, these regions had different electrophysiological characteristics. Region 3 had an activation profile consistent with normal propagation in repolarised tissue with an estV_m_ −57 ± 7 mV and an upstroke dF/dt_max_ 84 ± 12% of normal. There was an inverse correlation between estV_m_ and upstroke dF/dt_max_ and (linear regression slope −1.12 ± 0.23). Dual V_m_-Ca^2+^ imaging also suggested normal excitation–contraction coupling (ECC) in this region (Supplementary material online, *Figure S6*). Region 1 remained relatively depolarised (estV_m_ −6 ± 6 mV) and exhibited a low dF/dt_max_ (13 ± 7% of normal). In this region, dF/dt_max_ was not well correlated with estV_m_ (linear regression slope −0.25 ± 0.58). In addition, V_m_ changes during the PVC had no associated Ca^2+^ transient (Supplementary material online, *Figure S6*), consistent with electrotonic V_m_ changes in this persistently depolarised region.

At the site of PVC induction (Region 2) activation was effectively synchronous (∼2.5 mm over 0.7 ms, i.e. CV >350 cm/s). The average upstroke dF/dt_max_ in this region was 42 ± 11% of normal (*P* < 0.0001 vs. Region 1 and Region 3) and intermediate values of estV_m_ were observed (-28 ± 8 mV *P* < 0.0001 vs. Region 1 and Region 3) with a negative association (linear regression slope −1.93 ± 0.98). A delay between V_m_ and Ca^2+^ could not be resolved during the PVC upstroke in this region (Supplementary material online, *Figure S6*), suggesting that normal ECC was not operational, as V_m_-Ca^2+^ delay is typically 6–8 ms in optical recordings from rabbit epicardium.^35^ These analyses suggest that during electrotonic triggering of R-on-T PVCs, regenerative currents are activated from voltages in the approximate range of −20 to −40 mV (corresponding here to dF/dt_max_ of ∼30–60% of normal). This, together with the V_m_-Ca^2+^ relationship, implicates activation of *I*_CaL_ as the predominant mechanism of PVC initiation in these experiments. We next tested this hypothesis with the addition of the L-type Ca^2+^ channel (LTCC) blocker nifedipine.

### 3.5 LTCC blockade abolishes VA

**Figure 4:**
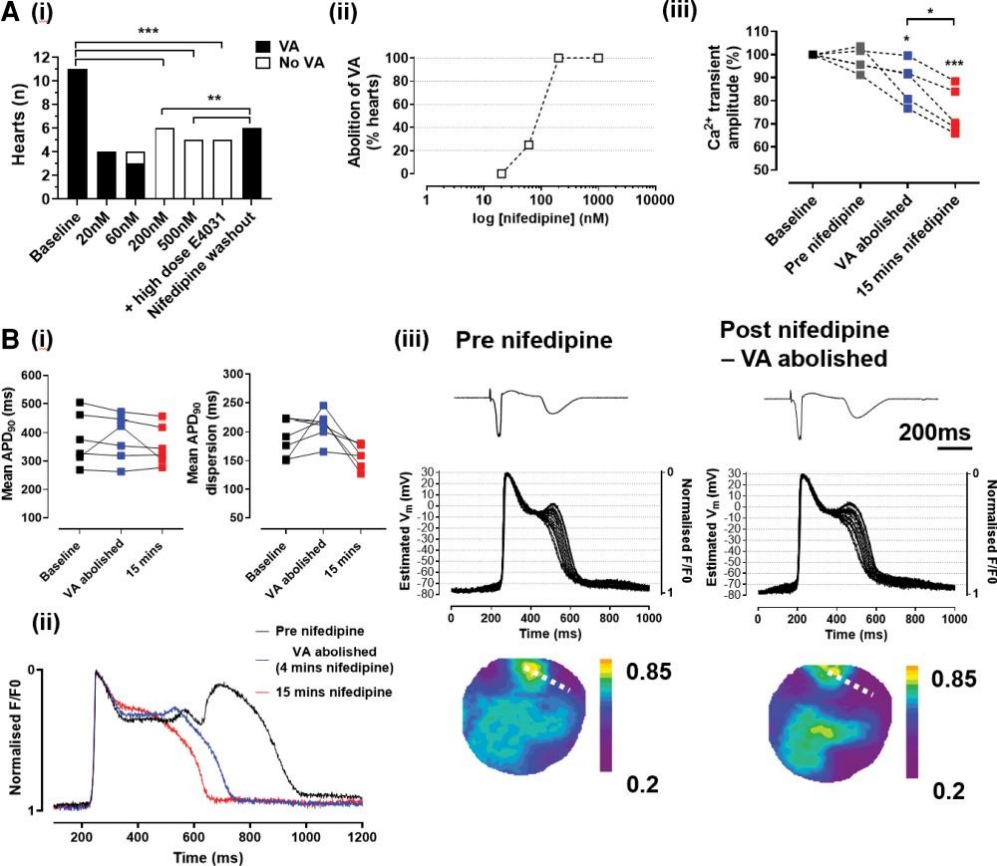
The effect of nifedipine on VA in aLQTS. (*A*) (i) Occurrence of VA, Fisher’s exact test ***P < 0.001; (ii) dose-response curves for the abolition of VA with nifedipine; (iii) effect of 200 nM nifedipine at baseline, immediately pre nifedipine, abolition of VA (0–4 mins) and after 15 mins, *n* = 5, RM ANOVA, ***P < 0.001, *P < 0.05 (vs. baseline unless otherwise indicated). (*B*) (i) Effect of 200 nM nifedipine on APD_90_ and APD_90_ dispersion at baseline, abolition of VA (0–4 mins) and after 15 mins perfusion, *n* = 6, RM ANOVA *P* = NS (ii) superimposed traces from the time points shown in B (i) and (iii) pseudo-ECGs, contiguous single-pixel V_m_ traces taken from across the border of the prolonged AP region (dashed line) plotted with estimated V_m_ and contour maps showing normalized F at the peak EAD before nifedipine (left panel) and after abolition of VA with nifedipine (right panel).

We studied the effect of low-dose nifedipine on the occurrence of LQTS-associated VA in 11 hearts. We used sub-maximal doses because we expected that larger doses would reduce Ca^2+^ transient amplitude, shorten APD and terminate VA indirectly.^24,25^ As shown in *Figure 4*, there was complete abolition of VA after the addition of either 500 nM (*n* = 5, *P* < 0.001) or 200 nM (*n* = 6, *P* < 0.001) nifedipine. In a subset (*n* = 5), we increased E4031 concentration (2µM) in the presence of 2–500 nM nifedipine but did not see a return of VA in any heart (*Figure 4Ai*). Washout of nifedipine resulted in a return of VA (*P* < 0.01 vs. 2–500 nM nifedipine). In a different subset (*n* = 4), we performed a dose-response protocol, quantifying VA occurrence over 10 minutes of 2000ms pacing during LQTS conditions and following the addition of 20, 60, and 200 nM nifedipine (*Figure 4A*). All four hearts had R-on-T PVCs at baseline; in one, these were completely abolished at 60 nM nifedipine and in the other three at 200 nM nifedipine. In all cases, abolition of VA occurred early during nifedipine perfusion (0–4 mins). We also observed a progressive reduction in PVC frequency with increasing doses of nifedipine (Supplementary material online, *Figure S7*).

The effect of nifedipine on Ca^2+^ transient amplitude was quantified (*Figure 4Aiii*). At the point of VA abolition, there was a modest reduction in the amplitude of the normalized Ca^2+^ transient (88.1 ± 9% of baseline, *P* < 0.05) as compared with the steady-state reduction at the same dose of nifedipine (75.4 ± 10% of baseline, *P* < 0.05 vs. VA abolition, *P* < 0.001 vs. baseline). The effective dose of nifedipine did not shorten APD or reduce dispersion in any heart (*Figure 4Bi*, APD_90_ 384.5 ± 89.2 ms vs. 375.3 ± 101.6 ms at baseline, APD_90_ dispersion 212.0 ± 29.0 ms vs. 181.1 ± 36.0 ms, *n* = 5, *P* > 0.05 for both). At the point of PVC abolition, islands of long APD, steep VGs and EADs were all still observed (*Figure 4Bii and iii*), confirming that the mechanism of the abolition of PVCs by nifedipine was not an indirect effect through modification of the LQTS substrate. These experimental data suggest a crucial role for *I*_CaL_ in initiating electrotonically triggered PVCs and that this mechanism is sensitive to low levels of LTCC block.

### 3.6 Threshold *I*_CaL_ required for electrotonic triggering *in silico*

**Figure 5:**
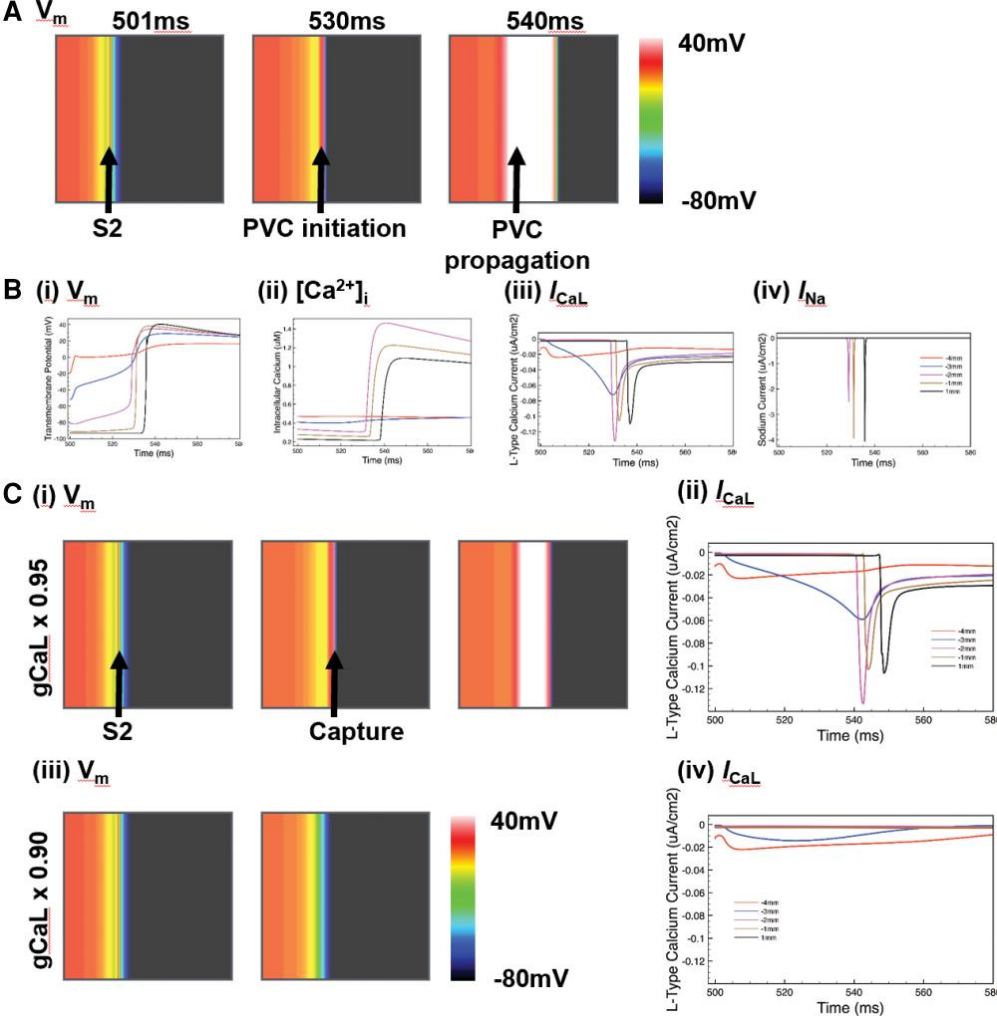
Role of *I*_CaL_ in electrotonic triggering in simulations. (*A*) (i) Colour plots of spatial V_m_ distribution from a simulation, showing the pseudo-EAD (S2) with subsequent initiation of a PVC at the border of the long APD region with propagation into the normal APD region. (*B*) Traces sampled at 1 mm spacing from the border between the long and short AP regions in A [red −4 mm, blue −3 mm, pink −2 mm, brown −1 mm, black +1 mm, negative values are towards the left of the map (within the long AP region) see Supplementary material online, *Figure S2*] for (i) V_m_, (ii) [Ca^2+^]_i_, (iii) *I*_Na_ and (iv) *I*_CaL_ from the application of the S2 until after the initiation of the PVC. (*C*) The effect of reduction of *I*_CaL_ on PVC initiation in simulations (i) V_m_ colour maps and *I*_CaL_ (ii) from a simulation identical to A(i), except for the reduction of gCaL by ×0.95. A PVC is initiated, with early activation of *I*_CaL_ at the border of the long AP region (blue trace, ii). V_m_ colour maps (iii) and *I*_CaL_ (iv) from a simulation in which gCaL has been reduced by ×0.9 in the model. In this case, there is no activation of *I*_CaL_ following S2, and no PVC is initiated.

We investigated the mechanisms underlying these experimental findings in the computational model (*Figure 5*). A pseudo-EAD occurring within the long APD region raised the level of V_m_ locally. This increased local VGs, triggering electrotonic current flow and causing PVC capture at 530 ms. *Figure 5B* shows expanded upstroke traces for V_m_ and [Ca^2+^]_i_ from the simulations, along with corresponding current densities for *I*_CaL_ and *I*_Na_. In the earliest activated site (blue trace, *Figure 5B*), depolarisation occurred due to *I*_CaL_ without any contribution from *I*_Na_. *I*_CaL_ began to increase near the EAD immediately after it was applied, causing the corresponding elevation of V_m_ at the same time (red and blue traces of *Figure 5Bi*). *I*_Na_ did not contribute to depolarisation in the long APD region, with sites 2 mm to the left of the boundary showing negligible *I*_Na_ (*Figure 5Biv*). In downstream sites with shorter APD, *I*_Na_ had sufficiently recovered and could mediate propagation, shown by the larger *I*_Na_ currents (brown and black traces of *Figure 5Biv*). *I*_Na_-mediated propagation was confirmed in this region by the AP upstroke dF/dt_max_, which became faster at these sites (brown and black traces of *Figure 5Bi*). Correspondingly, APs regained a normal morphology and were associated with normal ECC (*Figure 5Bii*). As shown in Supplementary material online, *Figure S5B*, the same dynamic behaviour of V_m_, [Ca^2+^]_i_, *I*_CaL_ and *I*_Na_ were seen across the border of long and normal AP regions in the absence of a pseudo-EAD, where the PVC was triggered purely by the steep VG.

Using this computational modelling framework, it was possible to modulate *I*_CaL_ by reducing the maximum conductance of gCaL independently. As shown in *Figure 5C*, simulations recapitulated the experimental findings. Reducing gCaL to 95% of its default value throughout the entire tissue still allowed the initiation of a PVC (*Figure 5Ci and ii*). Reducing gCaL to 90% abolished capture of the PVC (*Figure 5Diii and iv*). For these small reductions in gCaL, negligible changes were seen in AP morphology, APD or VGs between long and normal APD regions (comparing V_m_ distributions between *Figure 5Ci and iii* along with *Figure 5Ai*). *Figure 5C*(ii) and (iv) show *I*_CaL_ traces for 95 and 90% gCaL, highlighting how such a small reduction in *I*_CaL_ prevents *I*_CaL_-mediated PVC initiation. Similar abolition of PVC capture was also seen in the case in which gCaL was only reduced regionally in the long APD region (results not shown).

## 4. Discussion

This study examined mechanisms of R-on-T PVCs which initiate TdP in a perfused-heart model of aLQTS in rabbit and complementary computational models. Our data provide novel insights that are fundamental to understanding arrhythmia mechanisms and have important translational implications. The key findings are (i) PVCs are not directly generated by EADs; (ii) PVCs are electrotonically triggered by steep VGs; (iii) activation of *I*_CaL_ mediates the initiation of electrotonically triggered PVCs, and (iv) this mechanism is sensitive to low levels of LTCC block which abolishes PVCs and TdP, in the continued presence of LQTS conditions.

### 4.1 Discrete regions of APD prolongation are bounded by steep VGs

A key feature of the arrhythmia mechanism we observed was the development of discrete regions of extreme APD prolongation under aLQTS conditions. These long APD islands were bounded by steep VGs separating them from the surrounding myocardium. These have been observed in iLQTS,^25^ aLQTS^21–24,29^ and in computational models.^36^ In our study and others, these regions were seen to arise dynamically, with PVCs arising from the borders. Our study specifically identified focal PVCs arising from the epicardium using strict timing criteria. In a sample of 31 mapped epicardial PVCs from 13 hearts, we saw consistent initiation from the border of long APD islands and propagation into the surrounding myocardium. Combined with the consistent observations in other studies, this quantitative approach strongly suggests that the development of long APD islands bounded by steep VGs are responsible for LQTS-associated arrhythmia.

### 4.2 Role of EADs in LQTS-associated arrhythmias in myocardial tissue

Phase 2 EADs occurred periodically within long APD islands in keeping with their APD-dependence. The occurrence of EADs inside these islands could be a source for triggered PVCs. Indeed, it has been suggested that such islands would allow spatiotemporal synchronization of cellular EADs, as EAD-prone cells would be relatively ‘shielded’ from source–sink influences.^22^ However, our data do not support a direct link between the occurrence of tissue-level EADs and the induction of PVCs, as the spatiotemporal relationship between the EAD and the PVC upstroke was not close (a few mm and tens of ms), and they occurred in very different estVm ranges (∼+5 mV vs. ∼−40 mV).

In contrast, the peak VG occurred in a similar estV_m_ range and within a few ms and ∼1 mm of the PVC upstroke, directly implicating steep VGs in PVC initiation in tissue under LQTS conditions. Based on these data, we hypothesized that EADs were not a requirement for LQTS-associated PVCs. Attempts to separate EADs and steep VGs pharmacologically were not successful. We used thapsigargin (TG, 2 µM) which did not abolish EADs, steep VGs or arrhythmia (Supplementary material online, *Figure S8*). At higher doses of nifedipine, both EADs and steep VGs were abolished. We therefore used computational modelling to separate EADs and VGs and test the hypothesis that PVCs could arise purely through electrotonic triggering in the absence of EADs.

### 4.3. Electrotonic triggering by steep VGs in tissue

We constructed an idealised 2D model in which a sheet of simulated cells with long APD but without spontaneous EADs was coupled to a sheet with normal APD. This generated steep VGs at the boundary and, above a threshold VG, electrotonic triggering of PVCs occurred. Brugada and Wellens^37^ first suggested this could occur between areas of normal and short APD during regional ischaemia. Three-dimensional simulations of regional ischaemia in human ventricles suggested that steep VGs lead to electrotonic triggering of EADs facilitating transmural re-entry.^38^ In cryoablated hearts with aLQTS, electrotonic triggering of focal activation was observed in association with EADs in the ∼1 mm surviving epicardial layer.^23^ Our data extend the experimental evidence for electrotonic triggering to the intact heart and demonstrate that VGs alone can directly generate PVCs by this mechanism.

Despite the theoretical separation of steep VGs and EADs in the mechanism of PVC initiation we describe, there remained an association between the occurrence of EADs and steep VGs in our experiments. EADs occurring near the border of the prolonged AP region were responsible for a dynamic increase in local VGs, supported by the observation that only EADs close to the border of the long AP region were associated with PVCs. Our data suggest that steep VGs (which are in part generated by a local EAD), rather than the EAD itself, were responsible for activation of the regenerative inward current necessary for the PVC upstroke.

### 4.4 A key role for *I*_CaL_ in electrotonic triggering

Using high spatiotemporal resolution mapping of V_m_/Ca^2+^, we characterised electrophysiology in detail during PVC upstrokes to identify the currents responsible for electrotonic triggering. We show regions with distinct profiles compatible with: (i) passive current flow in the long APD islands (low upstroke dF/dt_max_ and CV); (ii) normal propagation in the repolarised myocardium (>85% normal), and (iii) a synchronously activated region, with upstroke dF/dt_max_ and V_m_ ranges compatible with *I*_CaL_ activation. These findings were confirmed in the computational simulations. Previous computational modelling studies have shown that PVCs can arise spontaneously from VGs when *I*_CaL_ is increased.^10,25,26^ Lui *et al*.^10^ employed detailed 3D simulations of genotype-specific LQTS hearts and also showed that PVCs arose dynamically from VGs in tissue, which they termed ‘R-from-T’ and were associated with TdP induction patterns similar to those seen in patients with LQTS. Their simulations required the presence of enhanced *I*_CaL_, but as in our modelling studies, and as suggested by our experiments, EADs were not a requirement for LQTS-associated arrhythmia. The congruent results across these two divergent modelling approaches underline the importance of *I*_CaL_-based electrotonic triggering in LQTS-associated arrhythmias and suggest that *I*_CaL_ may be a target for effective anti-arrhythmic therapy.

### 4.5 Role of *I*_CaL_ window current

Based on studies in isolated myocytes that identified a role for window *I*_CaL_ in the genesis of EADs, modifications of *I*_CaL_ window current in simulations have been demonstrated to terminate LQTS-associated arrhythmias.^10,25^ However, it is unclear how window current alone (as distinct from activation of the primary current) could support electrotonic triggering by VGs in the absence of EADs. The window current can generate a positive dV/dt only when the repolarising V_m_ tracks through the voltage range for the window current.^39^ In addition, reactivation of *I*_CaL_ can only occur after the E_m_ has been more negative than −40 mV for a sufficient time to allow recovery.^19^ We would argue that these two mechanisms are distinct in the PVC initiation we observe, because the window current helps maintain the regions of myocardium at positive potentials, while the reactivation of *I*_CaL_ is promoted by the negative voltages within the VG zone. Our data suggest that a very small reduction of peak current (5–10%) is sufficient to terminate LQTS-associated arrhythmia. Further work is required to establish how the primary current and window current may independently modulate PVCs generated electrotonically.

### 4.6 Electrophysiological basis for VG-mediated PVCs

One of the electrophysiological features of mammalian myocardium that underlies the PVC mechanism described is the well-established ability of *I*_CaL_ to support an AP in depolarised (−40 mV to −60 mV) ventricular tissue.^40,41^*I*_CaL_-dependent APs (Ca^2+^ AP) have a peak potential of approx. +10 mV and can propagate, albeit slowly, into more polarized regions to increasingly activate conventional Na^+^-current dependent APs.^42^ At membrane potentials more positive than −60 mV there is zero *I*_Na_ in the steady state and the time-constant of inactivation is ∼5 ms,^43^ so we would expect that at sites of PVC initiation (region 2) *I*_Na_ is completely inactivated. Based on this activation profile, *I*_Na_ will contribute to the AP in the V_m_ range (−60 to −70 mV), corresponding with region 3 in our study.

Another feature that favours the proposed mechanism is the background inward-rectifying potassium current that explains the tendency for the membrane potential to adopt a bi-stable (zero net current) state at either polarised (−80 mV) or depolarised (+10 mV) values.^44^ Therefore under certain circumstances, two comparably sized regions of differently polarised myocardium can exist for significant periods of time (100–200 ms).^45^ Finally, the size and extent of the zone between the polarised/depolarised regions, i.e. the region with the VG, is determined by the space constant of the tissue, which in mammalian tissue would predict a zone of 2.5–3.5 mm between the two polarized regions of myocardium.^46^ As demonstrated by computational modelling, if the magnitude and lifetime of the VG zone is sufficient, a Ca^2+^AP can be triggered within the VG zone that can propagate to a more polarised zone to initiate a PVC.^47^ The conditions to generate a PVC appears to occur in a discrete segment of the VG zone probably due to a nearby EAD event that generates a local large VG gradient sufficient to trigger a Ca^2+^ AP.

### 4.7 LTCC blockers as a therapeutic strategy in patients with iLQTS

While agents for specific pharmacological modification of window *I*_CaL_ are not available, our data suggest that low doses of LTCC block could be effective, particularly if the voltage-dependence of LTCC block could be optimised to target depolarised tissue. Published reports of LTCC blocker use in single cases^48–50^ and small series of patients,^51,52^ predominantly with iLQTS, have varied results. Most have used verapamil, which has *I*_Kr_-blocking activity at higher doses, at doses targeted at QTc shortening. Importantly, our data demonstrate that QTc shortening is not required for an anti-arrhythmic effect. As shown in Supplementary material online, *Figure S9*, on average complete abolition of VA occurred at a dose below the EC50 for LTCC block in cardiomyocytes.^53^

### 4.8 Limitations

#### 4.8.1 Relevance of the experimental model

There are inherent limitations to the use of animal models for the study of human disease, and our results should be interpreted in that context. However, the experimental model is well-established and characterised by spontaneous bradycardia-dependent VA^21–24,30^ a pattern seen in patients with both iLQTS and aLQTS. In experimental aLQTS, iLQTS^25^ and in patients, TdP is initiated by R-on-T PVCs, suggesting that a common arrhythmia initiation mechanism exists across species and models.

#### 4.8.2 Origin of PVCs mapped from the epicardial surface

Not all PVCs were mapped, and due to the limited depth resolution of the wide-field mapping we employed, we restricted our analysis to PVCs initiated close to the epicardial surface. This means that our study does not consider the contribution of M-cells or Purkinje fibres to PVC initiation. Other studies have used endocardial cryoablation to ensure that optical APs originated from the ∼1 mm of surviving epicardial myocytes. They also observed long APD islands with PVCs arising from the border.^23^ Choi *et al*.^21^ compared EAD events and TdP inducibility between cryoablated and non-cryoablated hearts and found no change in the occurrence of EADs or inducibility of TdP, providing support for the conclusion that transmural gradients, M-cell activity and/or Purkinje fibres are not pre-requisites for TdP in LQTS. Our data suggest that steep VGs are the key event in generating focal PVCs. Steep transmural VGs have been shown previously in LQTS and may have been responsible for some of the PVCs with later epicardial activation. Similarly, apico-basal gradients of intrinsic cellular APD may be a source of VGs in tissue, as may dynamic behaviours,^54^ anatomical features and structural discontinuities.^55^ Our study does not identify the source of the steep VGs and the development of steep VGs in LQTS is an important area for further work.

#### 4.8.3 Focal and re-entrant mechanisms of TdP

Although we did not study the interplay between PVCs and TdP, based on published evidence, it seems reasonable to propose that critically timed PVCs will interact with a vulnerable substrate and induce re-entry. Indeed unidirectional conduction block was routinely observed where long APD islands were large, and this may provide both the trigger and substrate for TdP. The fact that we did not observe any TdP after PVCs were abolished with LTCC blockade argues strongly that all VA in this model arose through focal PVC-dependent mechanisms.

#### 4.8.4 Computational modelling

The purpose of the computational model we employed was to test the primary hypothesis concerning repolarisation gradients and the role of Ca^2+^ current in initiating an ectopic beat. It was not intended to fully recapitulate the complexity of the cellular environment nor the various features which influence electrotonic coupling in the intact heart and is not an attempt to model LQTS. The simplicity of our modelling approach allowed us to isolate and vary individual model parameters whilst controlling others and simultaneously investigating spatial variations in individual ionic currents throughout the regions of interest, not possible experimentally. This allowed the model to isolate the specific phenomena of electrotonic triggering at sites of steep VG and dissect the individual mechanism of action as being due to activation of *I*_CaL_.

### 4.9 Implications for risk stratification

The arrhythmia paradigm we present here suggests that consideration of the balance between *I*_CaL_ and repolarising currents may be an important factor in understanding the risk of TdP. This is a potential explanation for the observation that drugs with ‘balanced’ ion channel effects can prolong the QT interval but without an associated risk of TdP.^56,57^ Recent data demonstrating co-expression of KCNH2 and CACNA1C in humans underline the importance of the balance between these currents.^58^

## 5. Summary

Our experimental and computational data confirm that PVC initiation in the intact heart under LQTS conditions is not directly mediated by synchronized EADs but by electrotonic triggering from steep VGs at the border of long APD islands. EADs occur within the long APD islands and contribute to dynamic increases in the local VG. This is a crucial mechanistic distinction, presenting a novel and tractable therapeutic target distinct from AP prolongation or EADs. Our data demonstrate that the primary event in arrhythmia triggering is the activation of L-type Ca^2+^ current in a limited area of myocardium by electrotonic current; and we go onto to show that, as a consequence, the subsequent VA is highly sensitive to low doses of LTCC block, even in the continued presence of LQTS conditions. This suggests a novel approach to reducing arrhythmic risk in LQTS based on voltage-dependent LTCC blockers. Translational studies to test the effectiveness of this strategy are warranted. From a wider perspective, our data and those of others support the idea that pro-arrhythmia in LQTS is a tissue-level phenomenon that cannot be predicted from isolated cell behaviour alone.

## Supplementary material

Supplementary material is available at *Cardiovascular Research* online.

## Supplementary Material

cvac103_Supplementary_Data

## Data Availability

The data underlying this article will be shared on reasonable request to the corresponding author.
