# Supplementary material for: Initiation of ventricular arrhythmia in the acquired long QT syndrome
Source: Cardiovasc Res. 2022 Jun 21;119(2):465–76. doi: 10.1093/cvr/cvac103 (PMC10064840; doi:10.1093/cvr/cvac103)
Supplement: cvac103_Supplementary_Data [file cvac103_supplementary_data.docx]

SUPPLEMENTARY MATERIAL

Initiation of ventricular arrhythmia in the acquired long QT syndrome

# Detailed Methods

## Experimental studies

### Langendorff-perfused rabbit hearts

All procedures involving animals were undertaken per the UK Animals (Scientific Procedures) Act 1986 under Project Licence PP52544544. Male New Zealand White rabbits (n=17) were anaesthetised with an intravenous injection of pentobarbital sodium (50mg/kg) containing 1000IU of heparin. Hearts were rapidly excised and Langendorff-perfused at 37°C with oxygenated (95% O2, 5% CO2) modified Tyrode’s solution of the following composition (in mmol/L): NaCl 128.2, CaCl2 1.3, KCl 4.7, MgCl2 1.05, NaH2PO4 1.19, NaHCO3 20 and glucose 11.1 (pH 7.4 ± 0.05). Flow rate (25-35mL/min) was adjusted to maintain a perfusion pressure of 60-70mmHg. One leaflet of the mitral valve was carefully cut with sharp forceps inserted through the pulmonary vein to prevent solution congestion after the suppression of ventricular contraction. This also prevented acidification of the perfusate and ischemia in the left ventricle (LV). Two Ag/AgCl disc electrodes were positioned in the bath to record a pseudo electrocardiogram (pECG) analogous to a lead I configuration, which was digitised and recorded continuously (LabChart). Atrioventricular nodal (AVN) conduction was ablated by injection of 10% formalin (0.01 – 0.02mls) into the compact AVN. AVN ablation was confirmed by persistent dissociation of atrial and ventricular electrical activity on the pECG, and the ventricular rate was typically < 30bpm. Hearts were then mounted in a custom chamber for optical imaging, and bipolar platinum stimulating electrodes were positioned on the apical epicardium at the border between the right and left ventricles (RV and LV) for pacing (cycle lengths 350 – 2000ms).

### Dual optical mapping of voltage and calcium

Hearts were loaded with the fluorescent intracellular Ca^2+^ ([Ca]_i_) indicator Rhod-2 AM (Molecular Probes, Eugene, OR; 0.5ml of 1mg/ml in dimethyl sulfoxide [DMSO] containing 10% pluronic acid) and were subsequently stained with the voltage-sensitive dye RH237 (Molecular Probes; 50ul of 1mg/ml in DMSO). Blebbistatin (Tocris Bioscience, Ellisville, MO; 10umol/L) was used to eliminate motion artefacts during optical recordings. Wide-field optical imaging of epicardial transmembrane potential (V_m_) and [Ca]_i_ was performed with a dual Micam 05 complementary metal-oxide-semiconductor (CMOS) camera system on a THT macroscope (SciMedia, Costa Mesa, CA) with a 32mm x 32mm field of view resulting in a spatial resolution of 0.1024mm^2^/pixel at a sampling rate of 1kHz. The anterior epicardial surface, including the RV and LV, was illuminated by LED light sources centred at 530nm (LEX-2G, Sci Media, Ex 531±40nm) focused directly on the surface of the preparation. Emitted light was collected using a 0.63x objective lens (Leica, Japan) and split with a dichroic mirror at 660nm (Leica, Japan). The longer wavelength moiety containing the RH237 signal was long pass filtered at >715nm and focussed onto the first CMOS sensor for V_m_ imaging. The shorter wavelength moiety was bandpass filtered between 575±15nm and focussed onto the second CMOS sensor for intracellular Ca^2+^ imaging.

### Experimental protocols

See Supplementary Figure I. Optical recordings were taken at baseline during ventricular pacing at cycle lengths (CLs) of 350ms / 2000ms and during intrinsic rhythm (IR). Pharmacological LQT was induced by switching to Tyrodes’ solution containing 50% K^+^/Mg^2+^ and 0.5µM E4031 (n=13). In 11 experiments, the effects of nifedipine were studied (200nM, n=6, 500nM n= 5). In 4 of these hearts, a dose-response for nifedipine was performed (20, 60 and 200nM). In 5, an increase in E4031 concentration (2µM) in the presence of nifedipine was studied.

The role of SR Ca^2+^ cycling in the genesis of EADs and LQTS-associated arrhythmia was investigated in a subgroup of five hearts using the non-competitive inhibitor of SERCA, thapsigargin (TG). All five hearts were perfused with 50% K+/Mg2+ + E-4031 0.5μM at 2000ms PCL, and then 2μM of TG was administered for at least 20 minutes. The dose and timescale for inhibition of SR function were based on previous studies.^1^

### *Data analysis*

Ventricular arrhythmias (VA) were identified from pECG appearances, and the occurrence of PVCs, bursts (2-5 PVCs) and TdP (> 5 consecutive ventricular beats of varying morphology^2^) was quantified. QTc was determined from the measured QT interval and corrected using Bazett’s formula.

Optical data analysis was performed using custom analysis software (*Optiq*, Dr Francis Burton). Optical data were processed with a Gaussian spatial filter (radius 3 pixels) before analysis. For both APs and CaTs, activation time (AT) was determined as 50% between peak and baseline amplitude and rise time as the time from 10-90% of the upstroke. For APs, repolarisation time at 90% (RT_90_) was determined at 90% of baseline amplitude and action potential duration at 90% (APD_90_) as RT_90_ – AT. Epicardial dispersion of APD_90_ was calculated using the 5-95% range of APD_90_. The rate-of-rise of the AP upstroke during PVCs was defined as dF/dt_max_ expressed as a percentage of the dF/dt_max_ during the preceding AP. For PVCs, the earliest AT relative to the pECG QRS was calculated. Where the earliest epicardial activation time was ≥20ms pre-QRS were likely initiated close to the epicardial surface, and these PVCs were analysed in detail. Consecutive single-pixel traces were exported, and fluorescence levels (F) were normalised to baseline (F0), resulting in F/F0 values where 0 represents the baseline and one the peak. We then assumed that for a normal AP, the upstroke would span -80 to +30mV, so F/F0 values 0-1 were mapped to a scale of -80 to +30mV to give an estimated V_m_ (estV_m_) for AP events. Voltage gradients (VGs) across sampled traces were quantified as estimated mV/pixel.

Continuous variables are presented as mean ± SD. Comparisons between two groups of continuous data were made using a Student’s t-test, paired where appropriate, and categorical data using Fisher’s exact test. Comparisons between three or more groups were made using one-way analysis of variance (ANOVA), repeated measures where appropriate, with Bonferroni’s post-testing for multiple comparisons. P<0.05 was considered statistically significant.

## Computational modelling studies

In-silico investigations were performed to dissect the specific mechanisms of the experimentally-witnessed phenomena.

### Model setup

A monodomain representation of cardiac electrophysiology was used to simulate pacing protocols over a 2-D sheet of cardiac tissue of dimensions 20mm x 20mm with fibre orientation in the *x*-direction (Supplementary Figure IIA). The sheet was discretised into a triangular finite element mesh of resolution 200um. Cellular ionic dynamics were represented by the general mammalian LuoRudyII cell model^3^, with tissue conductivity initially assigned to experimentally determined values.^4^ Simulations were performed with the Cardiac Arrhythmia Research Package.^5,6^

### Electrophysiological properties

To replicate the experimentally-witnessed islands of prolonged APD, the left-half of the tissue was assigned a prolonged APD by modulating the repolarising potassium channels (*I*_Kr_, *I*_Ks_) via reductions in the respective maximum conductances (Supplementary Figure IIA). To control the specific repolarisation gradient formed between the long APD region (left) and the normal APD region (right), tissue conductivity was globally modulated. Different combinations of prolonged APD and modulated tissue conductivity were used throughout.

### Pacing protocol

The model was paced initially 100 times to reach steady-state. A single S1 beat was then simulated by pacing the entire lefthand edge of the tissue to initiate a planar wave propagating left-to-right. In a sub-set of simulations, an additional S2 was prescribed at -4mm from the vertical tissue centre-line (within the region of long APD), stimulating a 200um-thin vertical strip of tissue, as shown in Supplementary Figure IIB. The S2 stimulus was delivered at 500ms following the S1 at threshold strength for 2ms duration. The purpose of this S2 was to represent a prescribed pseudo EAD delivered at a controlled specific timing and location, facilitating a direct comparison between different subsequent simulations.

### Data analysis

V_m_, [Ca]_i_, fast sodium current (*I*_Na_) and the L-type calcium current (*I*_CaL_) were analysed from a series of points located across the centre-line separating the long- and normal-APD regions (Supplementary Figure IIA).

# Supplementary Figures & Figure Legends


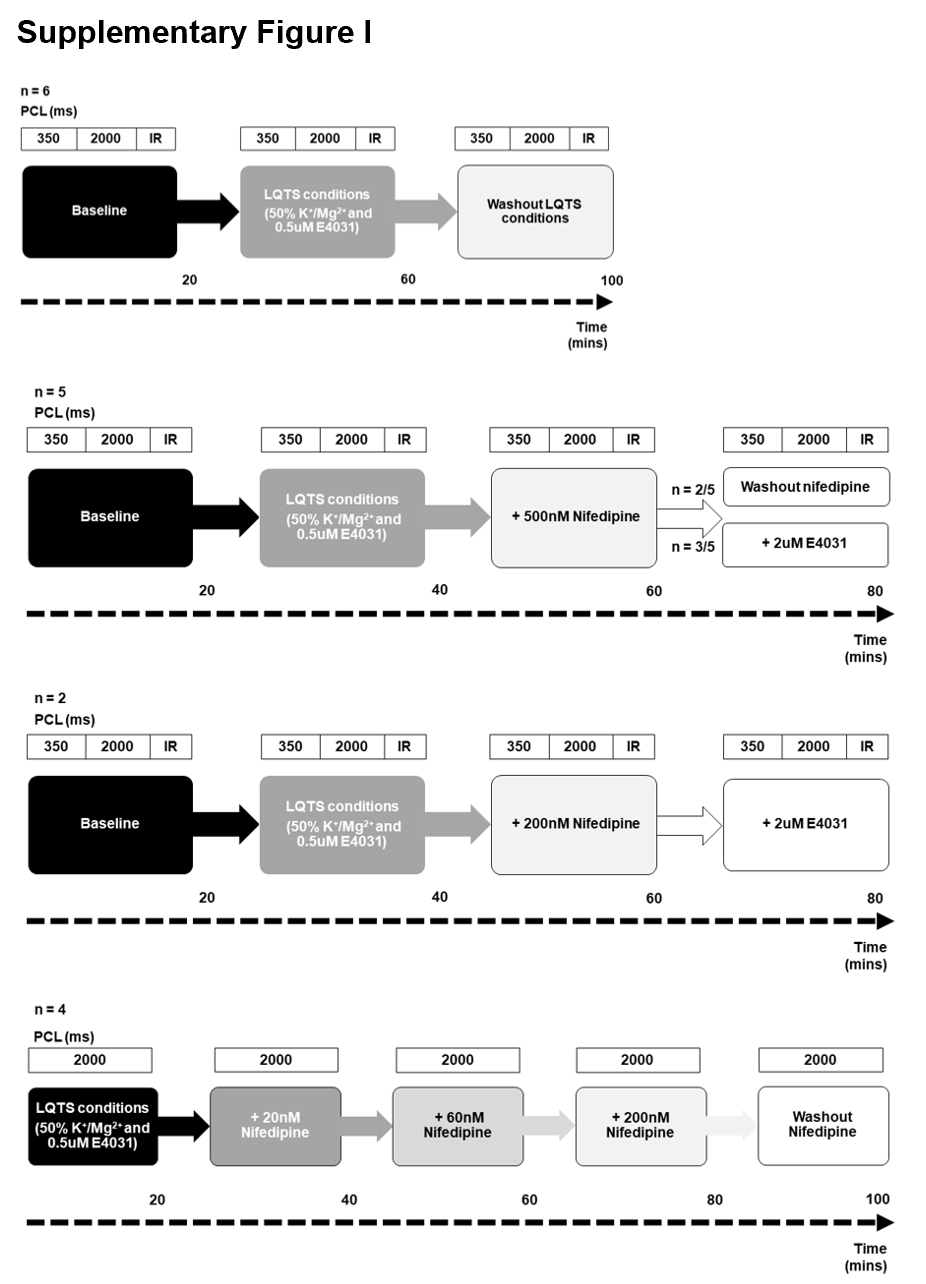


## ***Supplementary Figure I***

Diagrams to show the experimental protocols used. IR = intrinsic rhythm, LQTS = long QT syndrome, PCL = pacing cycle length.


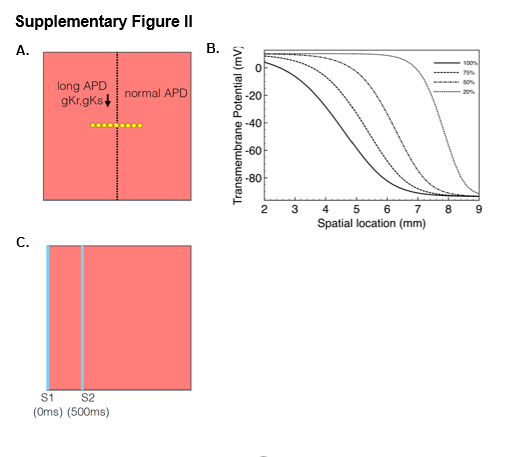


## ***Supplementary Figure II***

Schematic representations of model setup showing: A. prolonged and normal regions of tissue along with the recording sites used to analyse ionic currents and membrane potential (yellow dots). B. Spatial plots of V_m_ along the horizontal central line showing the transition from long to short APD regions, at t=600ms following the S1 at varying tissue conductivities. C.S1 and S2 stimulus locations, representing the initial paced beat and the simulated pseudoEAD stimulus, respectively.

##
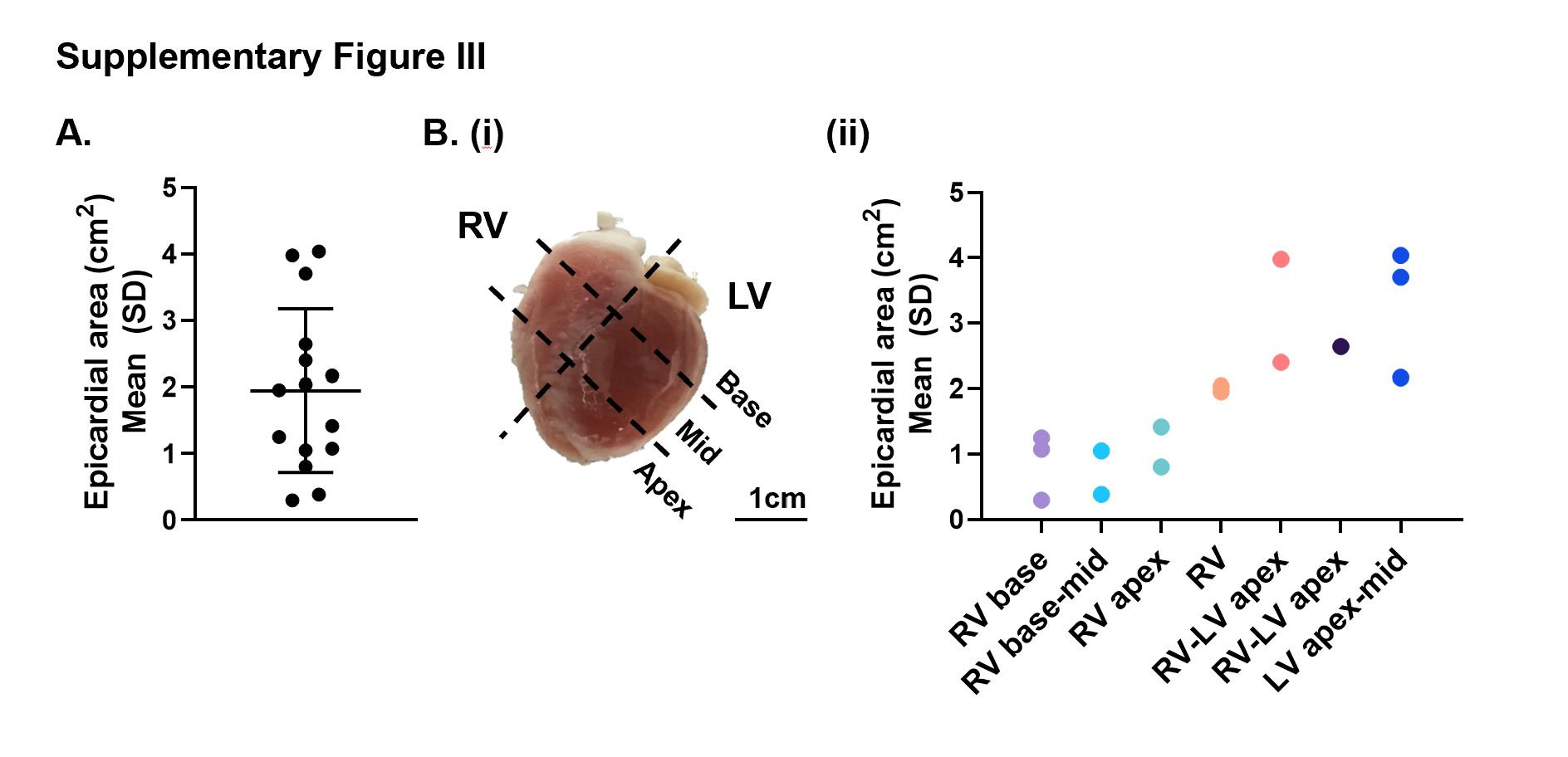
***Supplementary Figure III***

Supplementary Figure III. A. Epicardial area of APD islands during initiation of epicardial PVCs (n=15) during aLQT conditions in 7 hearts, mean 1.95±1.23cm^2^. B. (i) location definitions and (ii) epicardial area of APD islands stratified by heart and location.


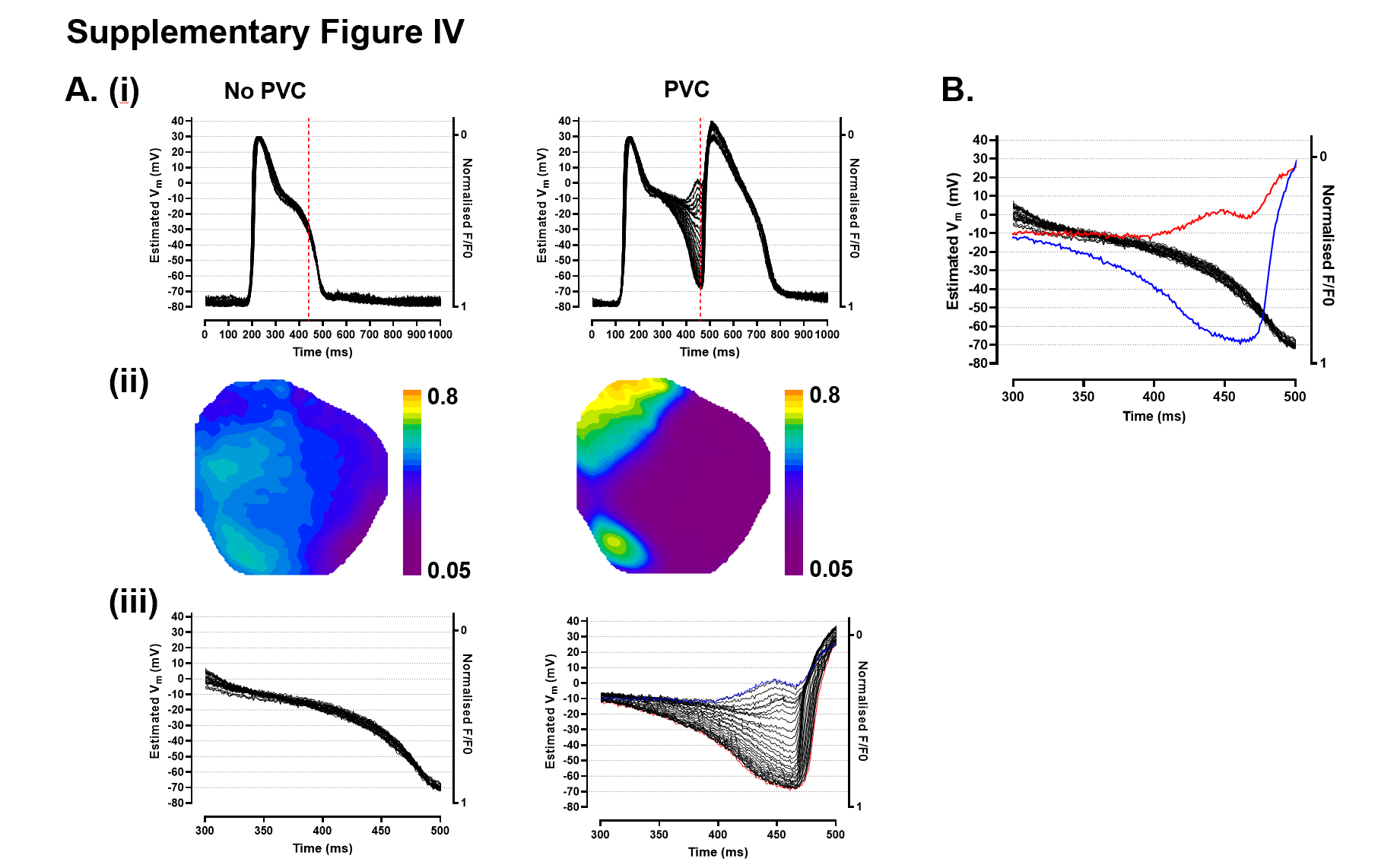


## ***Supplementary Figure IV***

A. (i) Contiguous single-pixel AP traces taken from white dashed line indicated in Fig 2A (i) and plotted with estimated V_m_ for beats with and without PVCs; (ii) contour maps showing normalised fluorescence (colour scale gamma 74) from the time indicated by the red dashed line in (i) with (iii) expanded plateau phases. B. Superimposed single-pixel plateau traces from A (iii) for the PVC beat the most depolarised (red) and least depolarised (blue) plateau traces are shown.


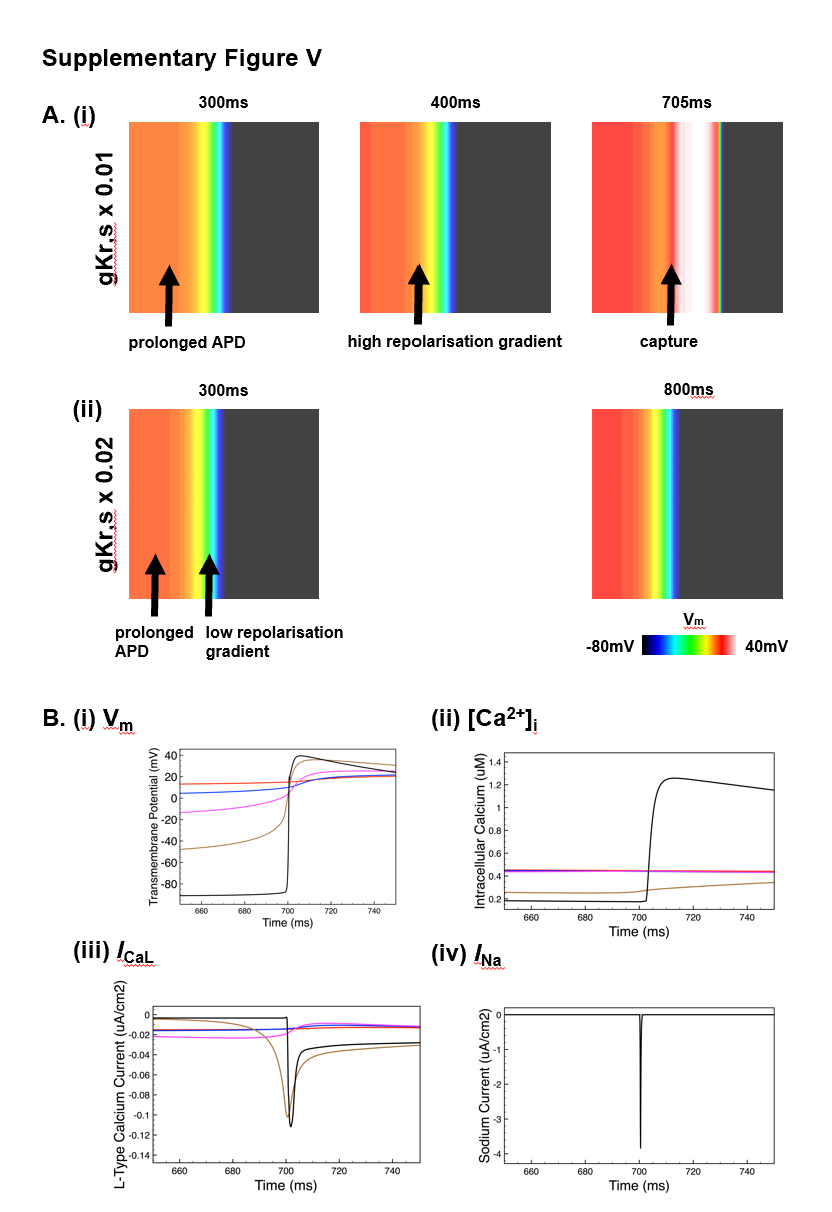


## ***Supplementary Figure V***

A. Spontaneous capture of tissue from long APD region driven by high repolarisation gradient. A) Snap-shots of spatial V_m_ distribution at different time instances following paced S1 beat in the case of a reduction in gKr,s to 10% (i) and 20% of normal values. In the former case, capture occurs at approximately 705ms, driven by a high gradient of repolarisation. In the case where the repolarisation gradient is lower (ii), no spontaneous capture occurs. B. Traces sampled at 1mm spacings from the border between the long and short AP regions in the simulation shown in A (red -4mm, blue -3mm, pink -2mm, brown -1mm, black +1mm, negative values are towards the left of the map [within the long AP region] see Supplementary Figure II) for (i) V_m_, (ii) [Ca^2+^]_i_, (iii) *I*_Na_ and (iv) *I*_CaL_ during the initiation of the PVC (~700ms).


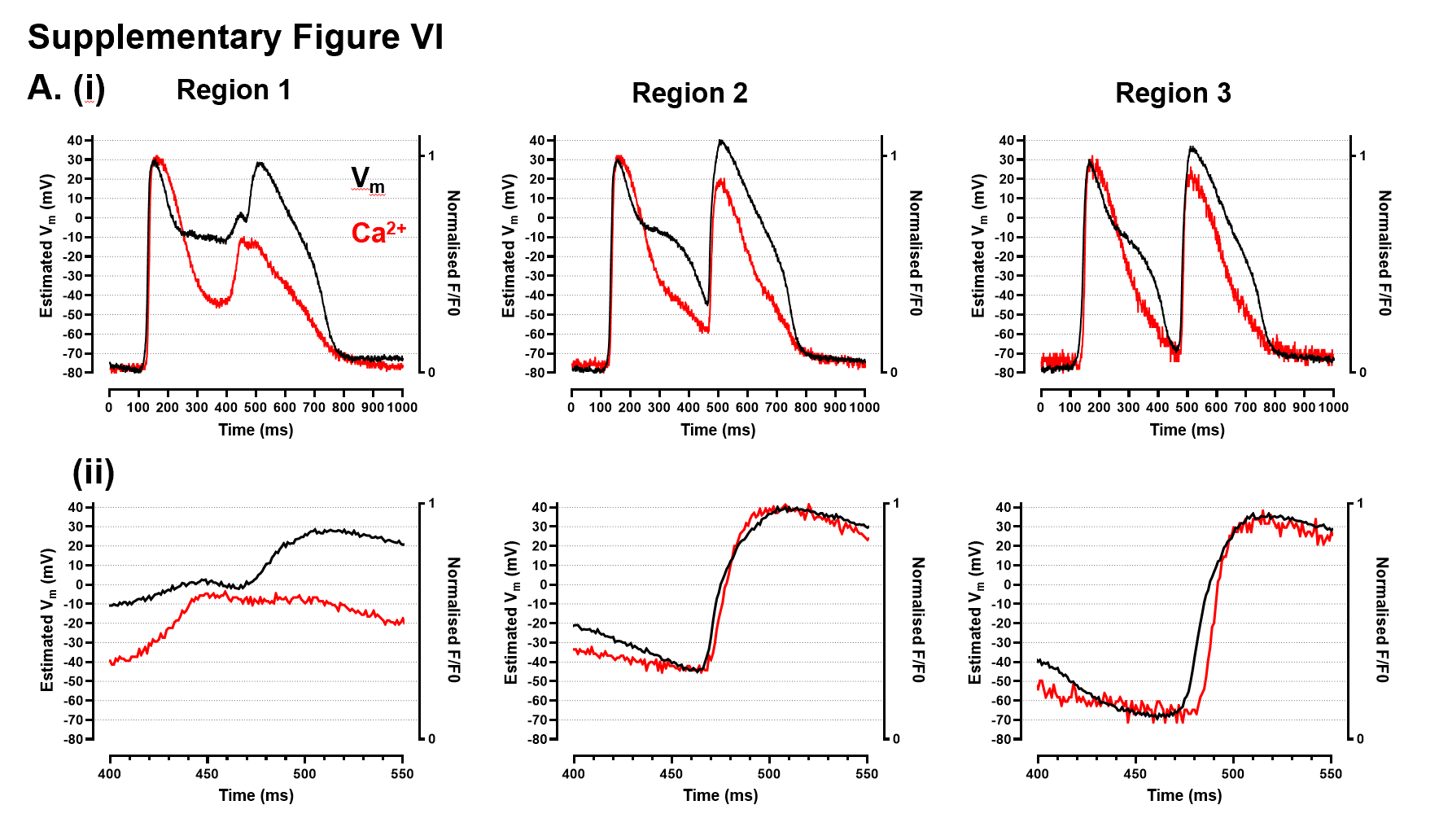


## ***Supplementary Figure VI***

Superimposed V_m_ (black) and Ca^2+^ (red) traces from representative points within each region with associated expanded upstroke traces shown in (ii). Region 1, within the long APD island, shows a Ca^2+^ -driven EAD followed by a V_m_ upstroke which is not associated with a Ca^2+^ transient. In region 2, at the earliest activation of the PVC, there is no EAD and V_m,_ and Ca^2+^ rise simultaneously during the PVC upstroke, indicating that normal EC coupling is not operating. In region 3, which contains propagation of the PVC in the repolarised myocardium, there is a normal V_m_- Ca^2+^ relationship during the PVC upstroke, indicating normal EC coupling.


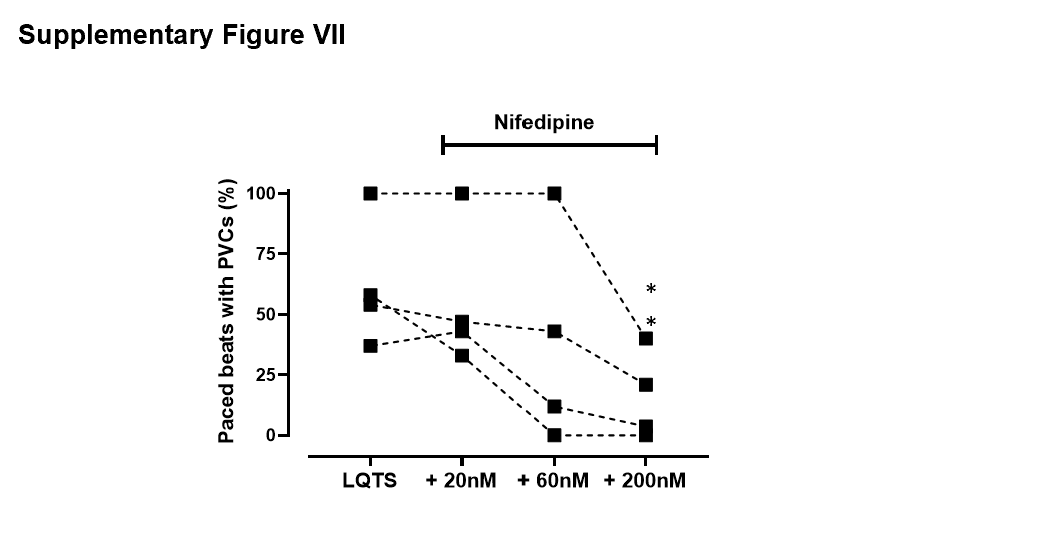


## Supplementary Figure VII

The proportion of paced beats with R-on-T PVCs under LQTS conditions with incremental doses of nifedipine (nM). Hearts were paced at 2000ms and observed for 10 minutes under each condition for quantification of PVCs. **repeated-measures one-way ANOVA, p<0.01 vs baseline.


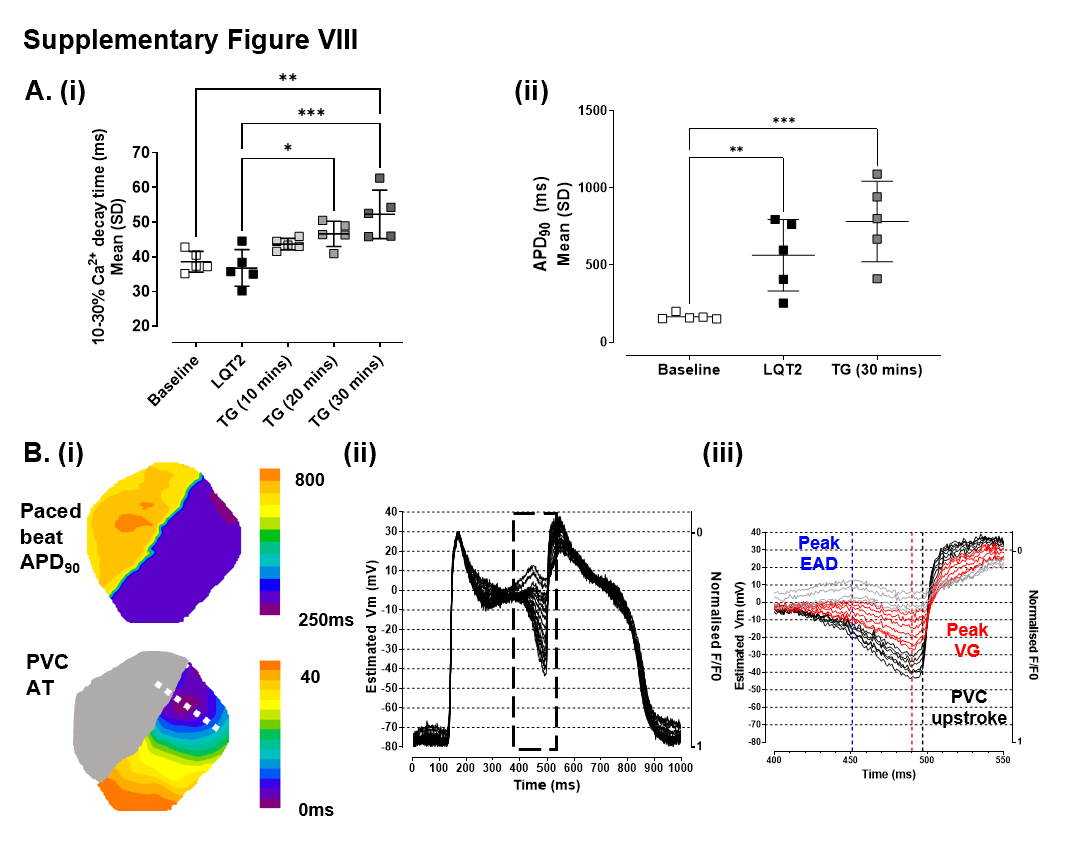


## Supplementary Figure VIII

The effect of SR inhibition with thapsigargin. A. (i) Mean values of 10-30% Ca^2+^ decay time for five hearts. 2µM thapsigargin (TG) significantly prolonged 10-30% Ca^2+^ decay time when compared with baseline at 20 minutes (46.6±3.6, p<0.05) and 30 minutes (52.2 ± 6.9, p<0.001), RM one-way ANOVA with Tukey’s post-testing; (ii) change in APD_90_ with TG. B. (i) Contour maps showing APD_90_ for a paced beat and activation time of the subsequent PVC arising from the border of the long AP island following 30 mins of TG, (ii) contiguous single-pixel AP traces taken from white dashed line indicated in (i) and plotted with estimated membrane potential (estV_m_); (iii) detail from (ii) expanded to show the relationship between the peak of the EAD (blue dashed line), the peak voltage gradient (VG, red dashed line) and the earliest upstroke of the PVC (dark grey dashed line). PVCs, bursts and TdP occurred in all five hearts tested before TG administration. After TG, there was no change in arrhythmia incidence (PVCs 5/5, bursts 5/5 and TdP 5/5, all p = NS). Analysis of PVC induction confirmed the same mechanism as seen pre-TG.


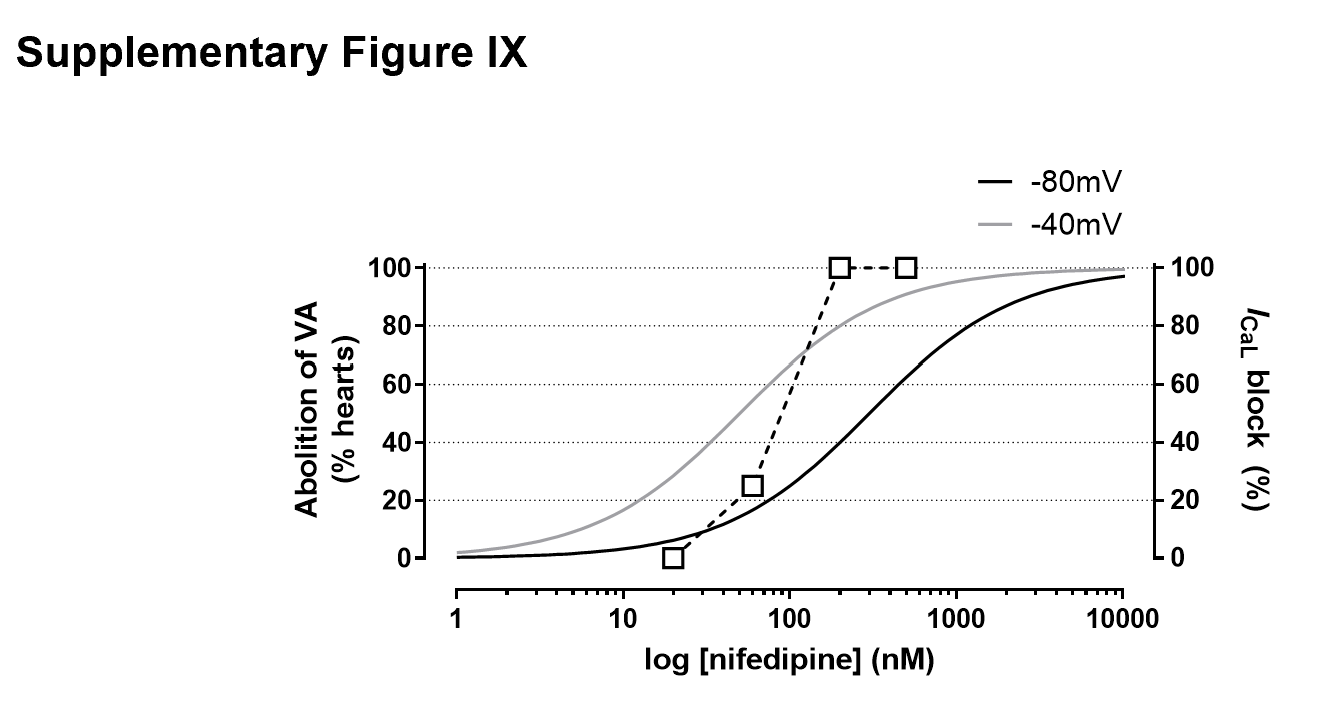


## ***Supplementary Figure IX***

Dose-response curves for nifedipine showing (i) proportion of hearts with the abolition of VA in these experiments (open squares, left y-axis) and (ii) percentage *I*_CaL_ block (right y-axis) in guinea pig ventricular myocytes at -80 (black) and -40mV (red) from Shen *et al.*^7^

# Supplementary Tables

## Supplementary Table I


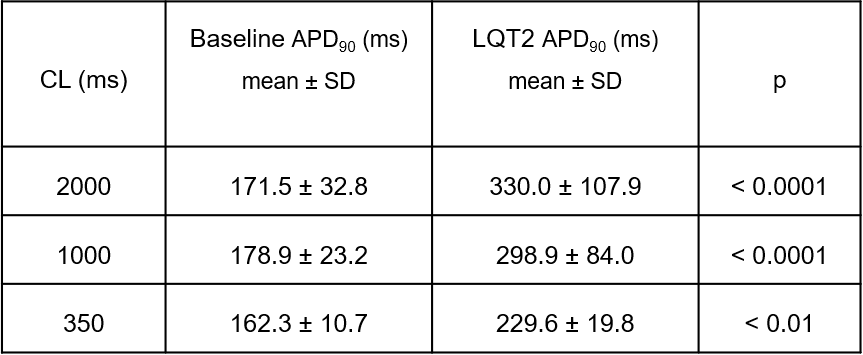


APD_90_ = action potential duration at 90% repolarisation, CL = cycle length, SD = standard deviation. n = 13, paired Student’s t-test.

# References

1. Elliott EB, Kelly A, Smith GL, Loughrey CM. Isolated rabbit working heart function during progressive inhibition of myocardial SERCA activity. *Circ Res*. 2012; **110**(12):1618-27.

2. Eckardt L, Haverkamp W, Borggrefe M, Breithardt G. Experimental models of torsade de pointes. *Cardiovasc Res*. 1998; **39**(1):178-93.

3. Luo CH, Rudy Y. A dynamic model of the cardiac ventricular action potential. I. Simulations of ionic currents and concentration changes. *Circ Res.* 1994; **74**:1071-1096.

4. Clerc L. Directional differences of impulse spread in trabecular muscle from mammalian heart. *J Physiol*. 1976; **255**(2):335–346.

5. Vigmond EJ, Hughes M, Plank G, Leon LJ. Computational tools for modeling electrical activity in cardiac tissue. *J Electrocardiol.* 2003;36:69–74.

6. Vigmond EJ, Weber dos Santos R, Prassl AJ, Deo M, Plank G. Solvers for the cardiac bidomain equations. *Prog Biophys Mol Biol.* 2008;96:3–18.

7. Shen JB, Jiang B, Pappano AJ. Comparison of L-type calcium channel blockade by nifedipine and/or cadmium in guinea pig ventricular myocytes. *J Pharmacol Exp Ther*. 2000; **294**(2):562-570.
